# 3D Printing of Resin Material for Denture Artificial Teeth: Chipping and Indirect Tensile Fracture Resistance

**DOI:** 10.3390/ma11101798

**Published:** 2018-09-21

**Authors:** Yoo-Jin Chung, Ji-Man Park, Tae-Hyung Kim, Jin-Soo Ahn, Hyun-Suk Cha, Joo-Hee Lee

**Affiliations:** 1 Division of Prosthodontics, Department of Dentistry, Asan Medical Center, 88 Olympic-ro 43-gil, Songpa-gu, Seoul 05505, Korea; wlsloo89@naver.com; 2 Department of Prosthodontics, College of Dentistry, Yonsei University, 250 Seongsanno, Seodaemun-gu, Seoul 03722, Korea; jimarn@yuhs.ac; 3 Division of Restorative Sciences, Herman Ostow School of Dentistry of University of Southern California, Los Angeles, CA 90089-0641, USA; thk@usc.edu; 4 Department of Dental Biomaterials Science and Dental Research Institute, School of Dentistry, Seoul National University, 101 Daehak-ro, Jongno-gu, Seoul 03080, Korea; ahnjin@snu.ac.kr; 5 Division of Prosthodontics, Department of Dentistry, Asan Medical Center, College of Medicine, University of Ulsan, 88 Olympic-ro 43-gil, Songpa-gu, Seoul 05505, Korea; chahyunsuk@hanmail.net

**Keywords:** 3D printing, CAD/CAM, resin, denture, fracture

## Abstract

3D printing of denture artificial teeth with resin materials is worthy of study in a novel way. This study evaluated chipping and indirect tensile fracture resistance of 3D printing resin material (Dentca 3D printing denture teeth resin) compared with conventionally prefabricated resin denture teeth (Premium-8, Surpass, SR-Orthosit-PE, and Preference). One hundred tooth specimens were prepared for testing. The 3D printed tooth specimens were printed at a 50 µm layer thickness with methacrylate-based photopolymerized resin by stereolithography 3D printing. Chipping and indirect tensile fracture tests were conducted at a speed of 1 mm/min until fracture. The indirect tensile fracture loads of the 3D printed resin teeth were higher than those of Premium-8, Surpass, and SR-Orthosit-PE, and lower than those of Preference teeth. Regarding chipping resistance, the 3D printed resin teeth were not different from Surpass and SR-Orthosit-PE, and were lower than Premium-8 and Preference teeth. The 3D printed resin teeth exhibited vertical fracture of the loaded cusp without deformation in chipping. The 3D printed resin teeth showed simultaneous fracture of two cusps in indirect tensile fracture, unlike other teeth. The results of this study suggest that 3D printing technology using resin materials provides adequate fracture resistance for denture artificial tooth use.

## 1. Introduction

Computer-aided design/computer-aided manufacturing (CAD/CAM) systems are widely used in dentistry, mainly for the fabrication of inlays, crowns, fixed partial dentures, and implant prostheses. Recently, CAD/CAM technology has been applied to fabrication of complete dentures, offering many advantages to dentists and patients over conventional complete dentures [1]. It allows a reduced number of appointments, and spare dentures are more easily available, as digital data are saved [2]. In addition, the laboratory work can be completed more conveniently and cost-effectively than by the traditional methods [3].

One of the pioneer CAD/CAM manufactured dentures for the treatment of edentulous patients is 3D printed dentures. The integration of digitalized modeling, computational optimization, and 3D printing procedures, suggests a novel method for denture design and fabrication [2]. The 3D printed denture uses additive technology to create a denture following digital design by complete denture process (Dentca denture; Dentca^TM^, Torrance, CA, USA). This 3D printing technology for dentures makes not only denture base, but denture teeth. These 3D printed teeth are made from methacrylate-based photopolymerized resin that is processed and cured by 3D printing only [4]. Denture artificial teeth and denture base are 3D printed separately, and then the printed teeth are bonded to the printed denture base using a light-cured bonding agent. Compared to traditional methods of making dentures by compression molding and hot water processing, 3D printing technology of manufacturing dentures is a new way and needs to be studied. If this technology is well studied and developed, 3D printed denture enables more efficient clinical adaptation which minimizes patient discomfort and potentially reduces long-term residual bone resorption [4]. Despite the many advantages of CAD/CAM dentures, if they are not well designed, they may show concerns, such as the short borders of denture bases and a lack of close tissue contact, which reduces retention [5,6]. Additionally, the mechanical properties of 3D printed denture teeth manufactured by CAD/CAM are, so far, unknown. 

Denture artificial teeth have been made chiefly from resin materials. The improvement of dental resin materials led to successful clinical application. The material grew from an inferior resin restorative, into the material of choice for both esthetic restorative treatment and removable denture prostheses [7]. This advancement was driven forward by improvements of the material, such as development of new monomers, filler technologies, and self-healing capacity [8,9]. One of the developments has focused on the filler systems, leading to improvements mainly in mechanical properties [10]. Composite resin with fillers was developed in an effort to achieve greater wear resistance than acrylic resin teeth [11], and to be less abrasive to human enamel antagonists than ceramic teeth [12,13]. Alternative monomers were adopted to reduce polymerization shrinkage and stress [14,15]. Recently, self-healing materials were introduced capable of restoring structure stability after damage happens [16]. Durability is an important property of dental materials, therefore, autonomous healing in oral cavities is highly desirable [9].

Denture artificial teeth in removable prostheses frequently fracture or chip [17,18]. In particular, where single complete denture opposes natural teeth or implant-supported overdenture, fracture or chipping of the denture teeth are very common complications [19]. In studies on implant-retained complete dentures in edentulous patients, the most frequent complication was fracture of the denture teeth [18,20]. It was also reported that at least one tooth had been changed due to fracture in 11% of the treatment cases for 10 years [21]. The current trend of using more implants in removable prostheses requires artificial teeth with high fracture resistance [1,22,23,24]. Many manufacturers have attempted to fabricate denture teeth with improved mechanical properties, including higher fracture resistance [19,25]. Resin materials provided increased fracture resistance and mechanical properties through the use of a higher degree of cross-linking between the polymers and the use of special pre-polymers (for example, Premium-8; Heraeus Kulzer GmbH, Hanau, Germany) [26]. The addition of inorganic fillers to the polymer matrix was used for the improvement of mechanical properties and wear resistance (for examples, SR-Orthosit-PE, Ivoclar Vivadent AG, Schaan, Liechtenstein; Surpass, GC Co., Tokyo, Japan) [27,28]. However, as fracture of denture teeth still remains problematic, there is a need for denture teeth manufactured using novel methods which can endure high load and force. Many studies on the fracture characteristics of dental restorative materials have been made on mode I fracture toughness, and studies on mixed mode bond strength have been actively conducted recently [29,30,31,32,33]. Dental restorations are subjected to combined shear and tensile loads due to forces from mastication [8,32]. Due to this complexity, some previous studies reported that traditional fracture criteria did not accurately predict the fracture results of dental materials and new methods such as extended maximum tangential strain criterion showed more accurate data predictions [34,35].

Since restorations fabricated and delivered at the dental clinics are not mass-produced but patient-customized, 3D printing manufacturing of denture artificial teeth is worthy, and this technology has received more attention. To confirm the clinical application and its effectivity of 3D printing material, it is necessary to investigate whether it has appropriate physical properties from a clinical point of view. Biomechanical characteristics of 3D printed denture teeth have not been reported yet, comparing those of conventionally manufactured denture teeth. Thus, it is required to evaluate clinical perspective fracture resistance of 3D printed denture teeth compared with conventionally manufactured denture artificial teeth used in dental field. The purpose of the present study was, therefore, to compare 3D printed denture teeth with conventional types of denture teeth, in regard to fracture resistance of chipping fracture and indirect tensile fracture tests. The null hypothesis was that there would be no difference in the fracture resistance among the denture artificial teeth.

## 2. Materials and Methods

### 2.1. Computer-Assisted Designing and 3D Printing for Denture Tooth Specimen Preparation

**Figure 1:**
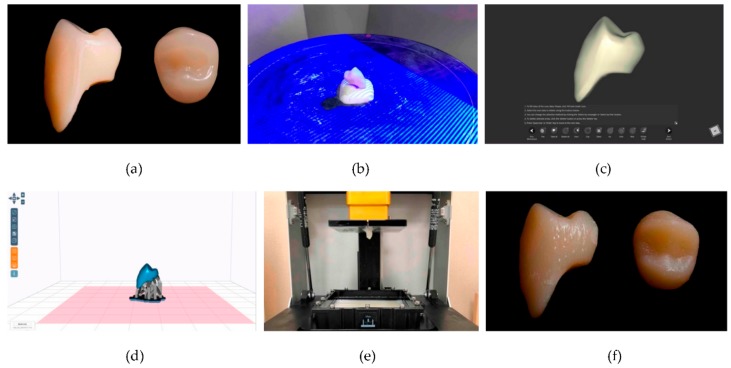
3D printed denture artificial tooth manufacturing procedure. (**a**) Prefabricated artificial denture tooth to replicate. (**b**) Original tooth was scanned with desktop scanner. (**c**) Shape of the scanned tooth. (**d**) Digital tooth file was loaded to 3D printer slicing software. (**e**) Tooth was 3D printed from the methacrylate-based photopolymerized resin material. (**f**) Printed tooth was cleaned and cured in the post-curing machine.

3D printed denture teeth and four different prefabricated denture teeth were used in this study. The 3D printed resin teeth were made of methacrylate-based photopolymerized resin (Dentca 3D printing denture tooth resin; Dentca^TM^, Torrance, CA, USA), according to the size and shape of the prefabricated denture maxillary first premolar tooth (Preference; Candulor AG, Glattpark, Switzerland) (Figure 1). 

Digital tooth file was uploaded to the 3D-printer software to prepare the pre-processing information for printing. The resin was poured into a 3D-printer (Zenith 3D-printer; Dentis Corp., Daegu, Korea), and teeth were printed layer by layer, also called stereolithography (SLA) technology. After being 3D printed at a 50 µm layer thickness, the teeth were cleaned with isopropanol and cured for a further 40 min by immersion into glycerin in the post-curing oven (UV Honle Sol 500; Honle UV America, Inc., Marlboro, MA, USA) to react the remaining monomers. As conventional types of prefabricated denture teeth, the maxillary first premolar teeth of Premium-8 (Heraeus Kulzer GmbH), Surpass (GC Co.), SR-Orthosit-PE (Ivoclar Vivadent AG), and Preference (Candulor AG) were selected for the current study (Table 1).

### 2.2. Chipping and Indirect Tensile Fracture Testing

**Figure 2:**
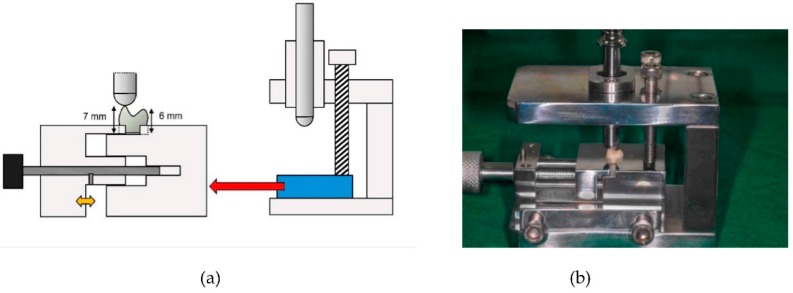
Chipping test. (**a**) Schematic drawing of the equipment manufactured for chipping experiment. The equipment was made into metal, and designed to apply loads to the buccal cusp tip of the denture tooth by point-to-point contact. (**b**) Photograph of a specimen placed for chipping test of this study.

Chipping and indirect tensile fracture tests were conducted to evaluate the strength from the clinical aspect of the prepared tooth specimens. Both tests were performed according to previous studies [24,36,37,38]. For the chipping test, equipment was designed so that the denture tooth specimen was located at a specific position, and did not move when receiving chipping force, prototyped by 3D printing and, finally, manufactured into metal (Figure 2). A pilot experiment confirmed that the tooth specimen was immobilized during the chipping experiment. The loading rod of the equipment contacting the denture tooth specimen had a hemispherical end that applied a load to the buccal cusp tip of the tooth by point-to-point contact. The bottoms of the denture teeth were ground to ensure a height of 7 mm from the base of the tooth to the buccal cusp and 6 mm to the palatal cusp, in order to apply the force only to the buccal cusp and to prevent the possibility of contact of the palatal cusp while loading (Figure 2a). The denture tooth specimen was secured to the equipment, which was mounted on a universal testing machine (Model 4465; Instron, Canton, MA, USA), and loaded at a speed of 1 mm/min. The load at which the chipping occurred was measured. The test was repeated 10 times for each type of the denture tooth.

**Figure 3:**
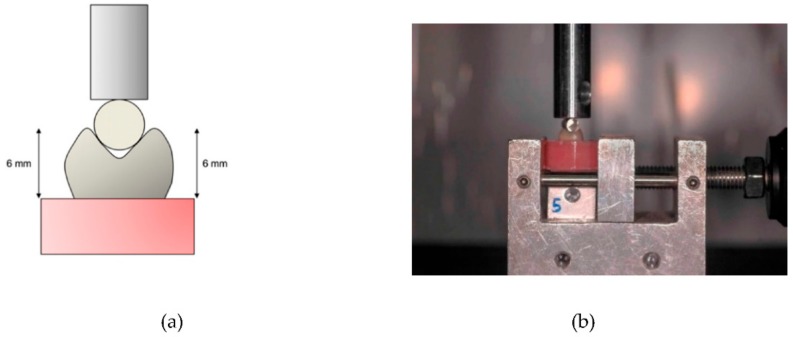
Indirect tensile fracture test. (**a**) Schematic drawing. Load was applied between cusps with a round metal bar to which an upper rod was connected. This was designed to apply force to surfaces at equal levels. (**b**) Photograph of a specimen placed for indirect tensile fracture test of this study.

The method used to measure indirect tensile fracture strength is shown in Figure 3. The prepared denture teeth were embedded with a self-polymerizing resin in cylindrical plastic molds. All embedded teeth in the specimens had a height of 6 mm from the base of the tooth to the buccal and palatal cusps, in order to apply the pressing force to surfaces at an equal level (Figure 3a). The specimen was positioned and secured to the assembly of the universal testing machine (Model 4465; Instron). A 4 mm diameter round metal bar was connected to the end of the loading rod of the machine, and fixed during loading (Figure 3b). The round bar was positioned to touch both cusp-slopes of the denture tooth. Load was applied at a speed of 1 mm/min until fracture occurred. The load at which the fracture occurred was measured. Ten samples were tested per each type of the denture tooth. 

### 2.3. Qualitative Analysis of the Fracture and Statistical Analysis

After each test, the fractured areas of the specimens were observed with field emission scanning electron microscopy (FESEM) (Hitachi S-4700; Hitachi, Ltd., Tokyo, Japan). The values from the groups were compared using one-way analysis of variance with Tukey’s honestly significant difference multiple comparisons test in SPSS (IBM Corp., New York, NY, USA). The significance level was set at *p* < 0.05. 

## 3. Results

The results of the chipping test are shown in Table 2. The load-to-chipping fracture values measured in Premium-8 (332.82 ± 54.64 N) and Preference (388.87 ± 55.64 N) teeth were significantly higher than those in the 3D printed resin (89.22 ± 14.87 N), Surpass (89.09 ± 33.75 N), and SR-Orthosit-PE (78.82 ± 25.99 N) teeth (*p* < 0.05). Preference teeth showed significantly higher chipping fracture resistance than Premium-8 teeth (*p* < 0.05). The 3D printed resin, Surpass, and SR-Orthosit-PE teeth did not differ significantly from each other. 

The values from the indirect tensile fracture test are shown in Table 3. The values of the load-to-tensile fracture was 160.28 ± 8.83 N for the 3D printed resin, 88.01 ± 29.05 N for Premium-8, 76.03 ± 13.38 N for Surpass, 98.29 ± 19.62 N for SR-Orthosit-PE, and 241.26 ± 26.34 N for Preference teeth. The values of the load-to-tensile fracture were significantly higher in the 3D printed resin and Preference teeth than those in Premium-8, Surpass, and SR-Orthosit-PE teeth (*p* < 0.05). Preference teeth revealed significantly higher tensile fracture values than the 3D printed resin teeth (*p* < 0.05). However, the tensile fracture values were not statistically significantly different among Premium-8, Surpass, and SR-Orthosit-PE teeth.

**Figure 4:**
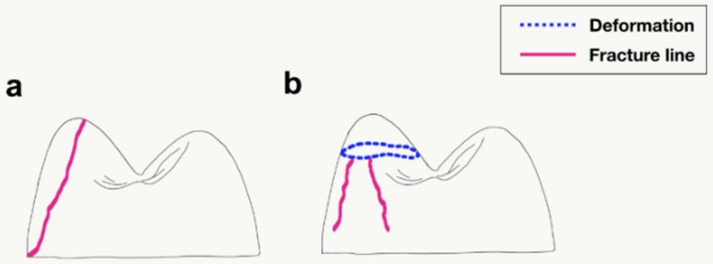
Two aspects of fracture after chipping test (sagittal view). (**a**) Fracture occurred from inner incline of cusp and spread downward along tooth wall. (**b**) Cusp tip was crushed and crack began around the periphery.

The failure patterns differed among the denture tooth types, and the fracture patterns showed a similar pattern within each test group. The fracture patterns after the chipping test consisted of two types (Figure 4). In 3D printed resin, Surpass, and SR-Orthosit-PE teeth, the fracture started from the inner incline of the loaded cusp near the loading point and progressed downward (Figure 4a). For the other, in Premium-8 and Preference teeth, the cusp was depressed and then the crack appeared at the periphery and spread downward (Figure 4b).

**Figure 5:**
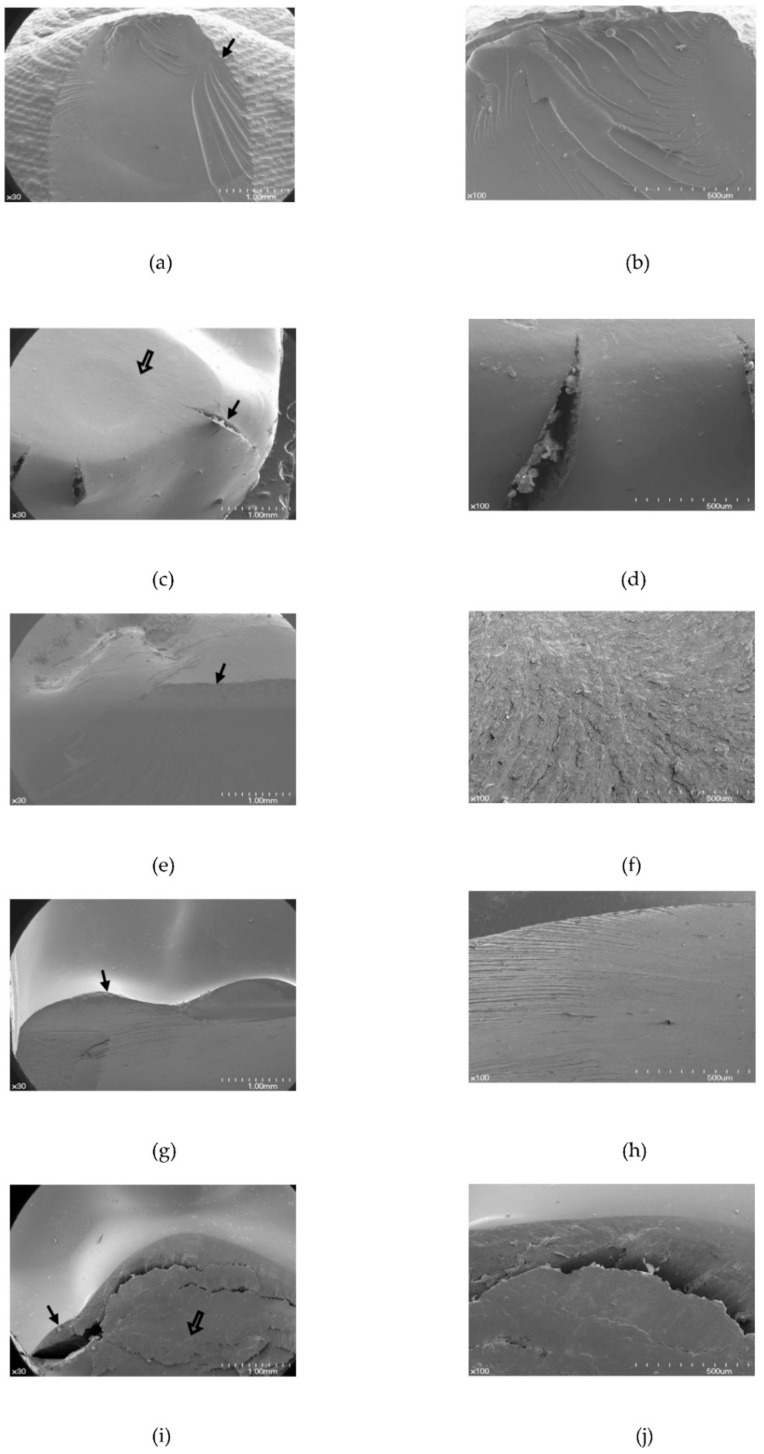
Scanning electron microscopy images after chipping test (occlusal view). (**a**) The 3D printed resin teeth (original magnification × 30); (**b**) the 3D printed resin teeth (original magnification × 100); (**c**) Premium-8 (original magnification × 30); (**d**) Premium-8 (original magnification × 100); (**e**) Surpass (original magnification × 30); (**f**) Surpass (original magnification × 100); (**g**) SR-Orthosit-PE (original magnification × 30); (**h**) SR-Orthosit-PE (original magnification × 100); (**i**) Preference (original magnification × 30); (**j**) Preference (original magnification × 100). Solid arrow designates fracture, and border arrow points to deformation.

Figure 5 shows FESEM images of the fractured denture teeth after the chipping test. Vertical fracture or fracture lines initiating from the buccal cusp were seen in the 3D printed resin, Surpass, and SR-Orthosit-PE teeth, which showed a buccal chipping fracture of the loaded cusp (Figure 5a,e,g). In Premium-8 and Preference teeth, there was initial deformation, such as a reverse cone-shaped depression, after which fracture occurred around the loaded buccal cusp (Figure 5c,d,i,j).

**Figure 6:**
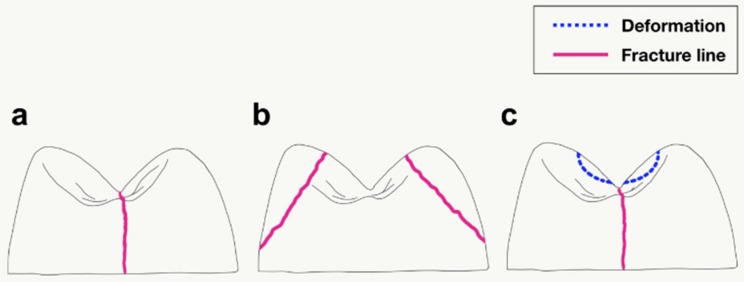
Three aspects of fracture after indirect tensile fracture test (sagittal view). (**a**) Fracture occurred in the middle of the tooth. (**b**) Tooth fragments fell off from both cusps near loading point. (**c**) After depression due to metal round bar, a center crack began and spread downwards.

The fracture patterns after the indirect tensile fracture test consisted of three types (Figure 6). The denture tooth types manifested different failure patterns, and each sample of a denture tooth type exhibited a similar fracture pattern after the indirect tensile test. In Premium-8, Surpass, and SR-Orthosit-PE teeth, the fracture went down along the center of the tooth (Figure 6a). In the 3D printed resin teeth, the fracture started from the inner incline of both cusps near the loading point and spread downward toward cervical area (Figure 6b). In Preference teeth, fracture occurred in the middle after the dent of the round bar shape (Figure 6c).

**Figure 7:**
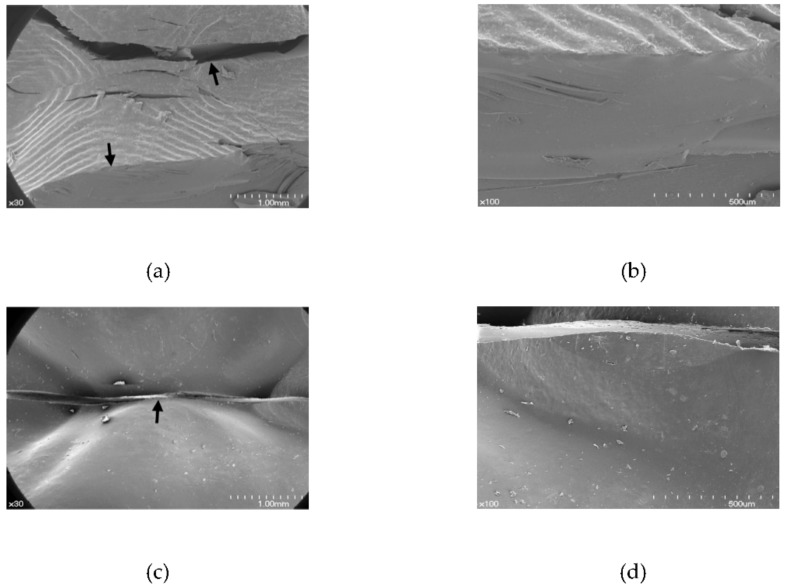

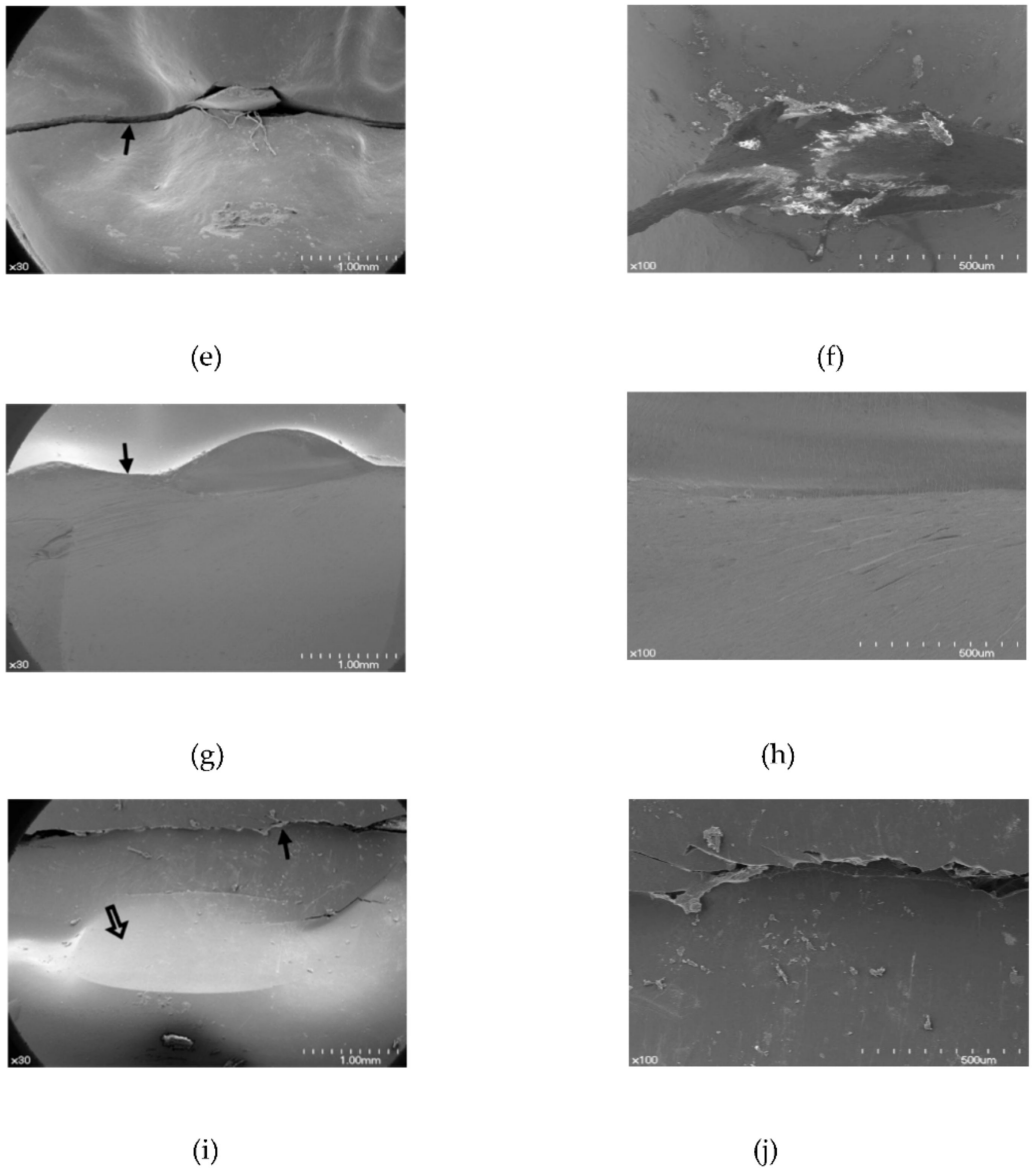
Scanning electron microscopy images after the indirect tensile test (occlusal view). (**a**) The 3D printed resin teeth (original magnification × 30); (**b**) the 3D printed resin teeth (original magnification × 100); (**c**) Premium-8 (original magnification × 30); (**d**) Premium-8 (original magnification × 100); (**e**) Surpass (original magnification × 30) (**f**) Surpass (original magnification × 100); (**g**) SR-Orthosit-PE (original magnification × 30); (**h**) SR-Orthosit-PE (original magnification × 100); (**i**) Preference (original magnification × 30); (**j**) Preference (original magnification × 100). Solid arrow designates fracture, and border arrow points to deformation.

FESEM images of the denture teeth after the indirect tensile test are shown in Figure 7. The 3D printed resin teeth showed a fracture in which buccal and palatal cusp of the teeth samples were both broken, rather than a fracture along the central groove of the tooth (Figure 7b). By contrast, a fracture line was seen along the central groove of Premium-8, Surpass, and SR-Orthosit-PE teeth (Figure 7c,e,g). In Preference teeth, deformation, such as a round-shaped depression, occurred initially, after which the tooth fractured along the central groove, similar to the fractures in Premium-8, Surpass, and SR-Orthosit-PE teeth (Figure 7i,j).

## 4. Discussion

The null hypothesis was rejected because the results according to the denture tooth types differed significantly. Additionally, fracture modes differed among the denture tooth types, and influenced fracture resistances significantly. Two damage modes were identified: fracture without distinct deformation; and fracture after deformation. Various contact responses to loading indenters have been reported, from the essentially “brittle mode” (crack-dominant) to the essentially “quasi-plastic mode” (deformation-dominant), which represent the microstructure inherent in the material [39].

In the chipping test, Preference and Premium-8 teeth showed high fracture strength. These two types of denture teeth showed deformation, such as cone-type depression, before fracturing. Figure 5 shows that, after the chipping fracture test, visible quasi-plastic deformation occurred with well-developed fractures. In the Premium-8 specimen (Figure 5c,d), a surface depression was seen, and corresponding subsurface cracks were apparent. In the Preference specimen (Figure 5i,j), the quasi-plastic deformation, at the surface and below the surface, became more apparent. However, the other three types of denture teeth, including the 3D printed resin teeth, did not show this pattern. 

In the indirect tensile fracture test, the 3D printed resin and Preference teeth showed high fracture strength, with Preference specimens showing quasi-plastic deformation before the fracture. The 3D printed resin, Premium-8, Surpass, and SR-Orthosit-PE teeth did not show quasi-plastic deformation. In the indirect tensile test, the fracture pattern of the 3D printed resin teeth was different from other teeth. Simultaneous fracture of buccal and lingual cusps was observed, rather than central line fracture that occurred in other denture tooth types. There was also no quasi-plastic depression pattern before fracture in the indirect tensile fracture test. Nonetheless, the fracture strength of the 3D printed resin teeth was as high as that of the teeth with quasi-plastic depression. This may be because other denture teeth are formed from a combination of different materials, such as enamel material, dentin material, etc., while the 3D printed resin tooth is made entirely of the same material by 3D printing.

In this study, the denture teeth with a quasi-plastic deformation pattern prior to fracture had higher fracture resistance than those without this pattern. The result seems to be due to the deformed shape of the teeth before they broke, which could withstand the load for a longer period of time. In a previous chipping fracture test of conventional denture tooth materials, large deformations were noted in soft materials [40]. In that study, the material with the highest hardness showed the weakest edge-chipping resistance. Bulging deformation and initial tooth split before the main piece chipped off were also exhibited; this was similar to the results of the present study. Characteristically, stiff materials exhibited a more sudden loss of strength than the quasi-plastic materials [39,41]. 

Ceramic denture teeth are now rarely used; however, denture teeth or bases still chip or fracture [21]. Fracture and separation of the denture teeth are also a frequent complication in other types of removable prostheses, such as implant-supported prostheses [42,43]. Denture tooth fracture is related to various factors. The durability and strength of denture prostheses were influenced by the chemical composition of the teeth and the denture base [44]. In that study, fractures reportedly occurred in the denture tooth itself (cohesive fracture) rather than between the denture base and the tooth (adhesive fracture). Thus, fractures were mostly affected from fracture strength of the denture teeth themselves [22]. Denture teeth are mainly composed of polymethylmethacrylate (PMMA) or urethane dimethacrylate (UDMA) resins. The minor components of each type of denture tooth and the size and amount of the filler vary [45]. There have been few other studies on the biomechanical aspects of 3D printed resin teeth. Dentca 3D printing denture teeth resin is a material developed specially for additive manufacturing. 3D printing of denture teeth is a novel method and uses new materials, therefore, it is necessary to evaluate whether it can be used clinically. In this study, the 3D printed resin denture teeth were compared with prefabricated resin denture teeth currently used in the dental field. The results of this study showed that the 3D printed resin teeth had comparable fracture resistance to some of the conventional prefabricated denture teeth.

One of the goals of material development is to produce materials that are better able to withstand the environmental conditions applied. Effort was carried out in defining a practical and theoretically testing framework [8,46]. Different fracture tests, such as three-point or four-point bending, have been employed, so far. However, it was reported that the traditional fracture criteria without considering T-stress was not accurate for estimating the crack kinking angles [31]. The extended maximum tangential strain criterion, which takes into account the effect of T-strain as well as the singular strain terms, was suggested to obtain more accurate predictions for the mixed fracture mode of dental restorative materials [34,35,47]. To investigate the physical properties of dental restoration, it would be better to measure fracture energy. If 3D printing technology develops and multi-layer artificial teeth can be additively manufactured in the future, further elaborate study is required to carry out through fracture energy analysis. Previous studies have compared the mechanical properties of various types of conventional denture teeth [20,22,36,40,42,48,49]. However, the various tests did not provide a full description of the fracture of denture teeth [50]. The chipping fracture and the indirect tensile tests used in this study were previously used to examine the mechanical strengths of denture teeth. In chipping experiments, the load was applied directly onto the cusp of tooth with an indenter using a universal testing machine, and the load at which chipping occurred was measured and compared [24,36]. Some quantitative indirect tensile fracture strength tests, in which force was applied directly onto buccal and lingual slopes of tooth, have also been described, and fracture strength (N) was recorded as the load at fracture [36,37,38]. Additional material studies are required on the physical properties of dental restorations manufactured by 3D printing with CAD/CAM. Further studies are needed to determine whether mechanical properties of the 3D printed resin teeth can support long-term oral functions to verify the long-term usefulness of the 3D printed teeth. Dynamic cyclic loading with suitable testing devices is also needed to prove the biomechanical stability of the 3D printed resin artificial teeth in the future.

## 5. Conclusions

3D printing combined with CAD/CAM methods can automate fabrication of dentures. The 3D printing technology for dentures makes not only denture base but denture teeth. Chipping strength and fracture resistance of the 3D printed resin artificial teeth should be established to address main complications associated with the dentures. The 3D printed resin teeth and four different prefabricated denture teeth were compared through chipping and indirect tensile fracture tests. The prepared denture teeth showed differences in the chipping strength and the indirect tensile fracture resistances, but the 3D printed resin teeth had fracture resistance and biomechanical pattern comparable to the conventionally prefabricated denture teeth. Within the limits of this in vitro study, manufacturing denture teeth with resin materials by 3D printing can be applicable in a dental clinical context.

## Figures and Tables

**Table 1:** Resin denture artificial teeth investigated in this study.*

Product	Manufacturer	Composition	Detail
Dentca denture teeth resin	Dentca^TM^	Methacrylate-based photopolymerized resin	3D printing
Premium-8	Heraeus Kulzer GmbH	MPM-PMMA	Mold XS
Surpass	GC Co.	CLC-PMMA-OFC	Mold 30M
SR-Orthosit-PE	Ivoclar Vivadent AG	Isosit (UDMA and inorganic fillers)	Mold N4
Preference	Candulor AG	PMMA with cross-linking of the polymer chains	Mold 840

MPM, multiplex-polymer-matrix; PMMA, polymethylmethacrylate; CLC, crosslink complex; OFC, organic-inorganic filler complex; UDMA, urethane dimethacrylate. * According to the manufacturer’s information.

**Table 2:** Results of the chipping test.

Denture Artificial Tooth	Mean Fracture Load in Newtons (SD)
3D printed resin teeth	89.22 (14.87) ^a^
Premium-8	332.82 (54.64) ^b^
Surpass	89.09 (33.75) ^a^
SR-Orthosit-PE	78.82 (25.99) ^a^
Preference	388.87 (55.64) ^c^

Different superscript uppercase letters indicate significant differences between groups (ANOVA followed by post hoc comparison; significance level 0.05).

**Table 3:** Results of the indirect tensile fracture test.

Denture Artificial Tooth	Mean Fracture Load in Newtons (SD)
3D printed resin teeth	160.28 (8.83) ^a^
Premium-8	88.01 (29.05) ^b^
Surpass	76.03 (13.38) ^b^
SR-Orthosit-PE	98.29 (19.62) ^b^
Preference	241.26 (26.34) ^c^

Different superscript uppercase letters indicate significant differences between groups. (ANOVA followed by post hoc comparison; significance level 0.05).
